# Validation of the Chinese Version of the Revised Internet Gaming Cognition Scale among Adolescents in China: Maladaptive Cognitions as Potential Determinants of Internet Gaming Disorder

**DOI:** 10.3390/ijerph17010290

**Published:** 2019-12-31

**Authors:** Yanqiu Yu, Phoenix Kit-han Mo, Jianxin Zhang, Jibin Li, Joseph Tak-fai Lau

**Affiliations:** 1 Centre for Health Behaviours Research, School of Public Health and Primary Care, The Chinese University of Hong Kong, Hong Kong, China; 1155101049@link.cuhk.edu.hk (Y.Y.); phoenix.mo@cuhk.edu.hk (P.K.-h.M.); 2 West China School of Public Health, Sichuan University, Chengdu 610041, China; zhangjianxin955@163.com; 3 Department of Clinical Research, Sun Yat-sen University Cancer Centre, Guangzhou 510060, China; lijibinzd@126.com

**Keywords:** gaming disorder, maladaptive cognitions, psychometric properties, adolescent, China

## Abstract

Maladaptive gaming cognitions are important determinants of Internet gaming disorder (IGD). Based on a systematic review, a 4-factor Internet gaming cognition scale (IGCS) was previously developed and cross-cultural validation of IGCS is warranted. The present study assesses the validation of the IGCS and its revised version, the Chinese version of Revised IGCS (C-RIGCS), among adolescents in China. Altogether, 755 students were recruited from junior middle schools in Guangzhou and Chengdu, China. The psychometric properties of the C-RIGCS were assessed by using appropriate statistical methods. The 4-factor model of the original IGCS was not supported by confirmatory factor analysis (CFA). In the split-half sub-samples, exploratory factor analysis suggested a 3-factor model for C-RIGCS, which was confirmed by CFA. The C-RIGCS and its three subscales showed satisfactory internal reliability, test-retest reliability, content validity, and absence of ceiling and floor effects (except on one case). Besides, the C-RIGCS and its three subscales were significantly correlated with external variables including IGD, gaming time, impulsivity, and self-control, and perceptions that Internet gaming is the primary source of self-esteem and social acceptance. The C-RIGCS proposed a new 3-factor model that showed satisfactory psychometric properties. It can be applied to understand maladaptive gaming cognitions of adolescent IGD.

## 1. Introduction

Internet gaming has become growingly popular across age, sex, and culture [1,2]. Adolescents are particularly vulnerable to psychosocial and psychological problems associated with Internet gaming [3]. In 2013, the Fifth Edition of Diagnostic and Statistical Manual of Mental Disorders (DSM-5) considered Internet gaming disorder (IGD) a mental health disorder; its nine diagnostic criteria were similar to those of substance dependence [4]. In 2018, the 11th edition of the International Classification of Diseases and Related Health Problems (ICD-11) further included online and offline gaming disorder as a disease [5]. It is imperative to understand determinants of IGD.

In particular, maladaptive cognitions are determinants of addictive behaviors such as IGD [6], Internet addiction [7], and pathological gambling [8,9,10]. Maladaptive cognitions on gaming include two categories: gaming related cognitive distortions and potentially harmful beliefs about the self in relation to gaming [6,11]. Potentially effective behavioral interventions for reduction of IGD (e.g., cognitive-behavioral therapy [12,13]) need to modify related cognitions [14]. In literature, emerging research has attempted to identify various cognitions of Internet gaming that are factors of IGD [6,15,16], as exemplified by a recent systematic review of 36 such studies [6]. The review proposed a 4-factor model that summarized the domains of potential cognitive determinants of IGD [6]: (1) overvaluation refers to the beliefs about reward value and tangibility of Internet gaming (e.g., perceptions that gaming rewards are as important as anything else); (2) maladaptive rules as a domain refers to justification of playing Internet games despite negative consequences; (3) gaming for self-esteem refers to over-reliance on Internet gaming to meet self-esteem needs; (4) gaming for social acceptance refers to using Internet gaming as a way to gain social acceptance [6]. Based on these four domains, the authors developed the Internet Gaming Cognition scale (IGCS), which discriminated between highly engaged adolescent gamers with and without IGD [17]. Some previous studies reported specific cognitive factors (e.g., preoccupation or cognitive salience) of IGD, but such factors are not specific to Internet gaming [6,18] or were limited to a relatively narrow range of cognitions related to Internet gaming [19]. IGCS has the strength of including factors that are specifically related to Internet gaming. Furthermore, it has considered such cognitive factors comprehensively, as it was derived from a systematic review of cognitive factors that were related to Internet gaming. Thus, the model has hence good potential for improving understanding and interventions regarding adolescent IGD. Cross-cultural validation of IGCS is warranted.

The present validation study hence investigated the psychometric properties of the Chinese version of IGCS among adolescents in mainland China. Such properties included the factor structure, internal consistency, test-retest reliability, and correlations with some multi-dimensional external variables on IGD, gaming time, impulsivity, self-control, and perceived importance of Internet gaming as primary sources of one’s self-esteem and social acceptance. IGD and gaming time were included as external variables, as maladaptive cognitions were correlated with addictive behaviors. We expect those with strong impulsivity and/or weak self-control to be more likely than others to adopt maladaptive rules. Perceptions that Internet gaming being a source of self-esteem and social acceptance were expected to be associated with gaming for esteem and gaming for social acceptance.

## 2. Materials and Methods

### 2.1. Study Design and Data Collection

The survey was conducted from October to December of 2018 in Guangzhou and Chengdu, China. The cities are located in southern and south-western China and have 14.9 and 16.3 million permanent residents, respectively. Four conveniently selected junior middle schools from the former (Grade 7–8 classes) and three from the latter (Grade 7–9 classes) cities consented to join this study; all the students of the relevant grades were invited to participate in the study. The fieldworkers briefed the students about the purpose and logistic of the study. They highlighted the anonymous nature of the study, that the return of the completed questionnaire implied informed consent to participate in the study, that students could quit anytime without being questioned, and that there were no negative consequences for refusals. Such information was also printed on the cover page of the questionnaire that the students were requested to read. The students self-administered the anonymous structured questionnaire in the absence of teachers and in classroom settings. The field workers answered inquiries and cross-checked the completed questionnaires. Of the 773 completed questionnaires, 18 (2.3%) were excluded from data analysis because there were more than 20% missing data in one or more scales. The remaining 755 (97.7%) questionnaires were completed by 261 students in Guangzhou (34.6%) and 494 in Chengdu (65.4%), respectively. To gauge test–retest reliability, 48 Grade 8 Chengdu students completed the same questionnaire twice within a 2-week period. No incentive was given to students. The study was approved by the Survey and Behavioral Research Ethics Committee of the Chinese University of Hong Kong.

### 2.2. Measurements

#### 2.2.1. Maladaptive Gaming Cognitions

Such cognitions were assessed by the 24-item IGCS developed by King and Delfabbro [17]. As previously mentioned, it included four subscales [i.e., overvaluation (5 items), maladaptive rules (8 items), gaming for self-esteem (6 items), and gaming for social acceptance (5 items)]. Two bilingual researchers translated the English version into Chinese; another bilingual researcher performed back-translation independently. A panel of three other bilingual researchers who were experienced in adolescent mental health and addiction research reviewed the process and finalized the questionnaire. All items were rated on 5-point Likert scales (0 = never to 4 = always). Four respective summative subscale scores were constructed, with scores ranged from 0 to 96; higher scores indicated higher levels of maladaptive gaming cognitions.

#### 2.2.2. IGD

The present study used the 9-item DSM-5 checklist to measure IGD [4] as the ICD-11 definition was released only recently and comparable diagnostic and screening tools for IGD are still under development. A number of studies had used DSM-5 to define IGD [17,20,21]. The checklist recorded presence of addictive symptoms (preoccupation, withdrawal, tolerance, inability to control, loss of interest in other activities, psychological and/or social problems, deception, avoidance, and significant loss due to gaming); IGD is defined by endorsement of ≥5 items. The Chinese version has been validated with good psychometric properties and diagnostic validity [20,22], and applied to empirical studies [20,21]. The Cronbach’s alpha of the scale was 0.75 in the present study.

#### 2.2.3. Other Variables Related to Internet Gaming

Participants were asked whether they had played Internet games in the past 12 months and, if any, their average gaming time per week in the past month. Two items were created by the panel to evaluate the importance of Internet gaming in obtaining self-esteem and social acceptance (“Internet gaming is the primary source of my self-esteem.” and “Internet gaming is the primary source of my social acceptance”), with 5-point Likert scales (1 = extremely disagree to 5 = extremely agree).

#### 2.2.4. Impulsivity and Self-Control

Impulsivity was measured using the 10-item Motor subscale of the Barratt Impulsivity Scale (BIS), which indicates tendency to act on the spur of the moment and fast reactions [23]. The Chinese version made some cultural adaptations and showed good reliability and construct validity in Chinese adolescents [24]. A sample item is “I do things without thinking”. The Motor subscale was measured with 5-point Likert scales (1 = completely disagree to 5 = completely agree), with higher scores indicated higher levels of impulsivity. The Cronbach’s alpha of the scale was 0.91 in the present study. Self-control was measured by the 13-item Brief Self-control Scale (BSCS) [25], which also showed good psychometric properties in Chinese adolescents [26]. A sample item is “I am good at resisting temptation”. The BSCS was unidimensional; all items were rated with 5-point Likert scales (1 = never to 5 = always), with higher scores indicated higher levels of self-control. The Cronbach’s alpha of the scale was 0.74 in the present study.

### 2.3. Statistical Analysis

We performed confirmatory factor analysis (CFA) with maximum likelihood estimation to confirm the 4-factor structure of the published study [6,17]. Goodness-of-fit statistics and cut-off criteria were: Chi-square/df ratio < 5.00, both the Non-Normed Fit Index (NNFI) and Comparative Fit Index (CFI) > 0.90, and Root Mean Square Error of Approximation (RMSEA) < 0.08. If the goodness-of-fit of the CFA was unable to confirm the 4-factor structure proposed by King and Delfabbro [6], we would follow the recommendation and practice of some researchers [27,28,29], randomly split the sample into two halves, and then performed exploratory factor analysis (EFA) in one subsample for factor extraction (varimax rotation methods and retainment of factors with eigenvalue > 1.0) and CFA with maximum likelihood estimation in the second subsample to confirm the factor structure identified by EFA. In EFA, items with all factor loadings ≤ 0.4 was firstly removed. In a stepwise manner, we removed double-loaded factors (i.e., those with factor loadings > 0.4 on multiple factors); the item with the heaviest factor loading was firstly removed and EFA redone; the removal process was repeated until no double loading remained. The method has been used in a number of published papers [30]. Floor and ceiling effects were present if more than 15% of the participants possessed the minimum or maximum scores of IGCS or its subscales, respectively [31]. Internal consistency was assessed by Cronbach’s alpha coefficients; test–retest reliability was evaluated by the Intra-class Correlation Coefficient (ICC). Item-scale and item-subscale Pearson correlation coefficients were derived. Besides, external validity was established by inspecting Pearson and Spearman correlation coefficients between the scale/subscale scores and the external variables. CFA was conducted by using AMOS 17.0 (IBM, Armonk, NY, USA) while the other tests were analyzed by SPSS 21.0 (IBM, Armonk, NY, USA). Statistical significance was defined as *p* < 0.05 (two-tailed tests).

## 3. Results

### 3.1. Participants’ Characteristics

Of the 755 participants, over half were males (53.1%); 11.6% perceived lower or much lower household income level compared with their fellow classmates; 21.5% self-reported below-average academic performance.

Among all participants, the prevalence of IGD was 11.7%; about two thirds (66.9%) had played Internet games in the past 12 months, and the prevalence of IGD was 16.2% among gamers; 14.7% had played Internet games more than six hours on average per week in the past month. About 7% perceived Internet gaming being the primary source of their self-esteem (7.7%) and social acceptance (6.7%), respectively. The mean scores for impulsivity and self-control were 22.8 (SD = 7.9, range: 5–50) and 44.2 (SD = 7.7, range: 5–65), respectively (see Table 1).

### 3.2. CFA for the Chinese Version of the Original IGCS

The results were presented in Table 2. Although the standardized path estimates that ranged from 0.46 to 0.77 were all statistically significant (*p* < 0.001), the 4-factor model yielded an unsatisfactory fit (Chi-square/df = 5.298; NFA = 0.870; CFI = 0.891; RMSEA = 0.076). In addition, the correlations among the four factors were very high (0.88–0.98). As mentioned in the Methods, we thus performed EFA and CFA in the two random sub-samples.

### 3.3. The Factor Structure of the Chinese Version of the Revised Internet Gaming Cognition Scale (C-RIGCS)

EFA was performed on the 24 items of the Chinese version of the original IGCS. Some revisions were made. Firstly, nine double-loaded items (i.e., items with factor loadings ≥ 0.4 on multiple factors) were removed through a stepwise process that involved removal of an item and repeated EFA in each step. The new EFA on the remaining 15 items yielded three factors (see Table 3) that showed eigenvalue > 1.0. [Factor 1 (7 items): Perceived rewards of Internet gaming that congregates items related to various types of perceived rewards expected from Internet gaming (e.g., feeling better, feeling more in control, feeling safer and more comfortable, and notice and respect from others). Factor 2 (4 items): Perceived urges for playing Internet games that contain items about eagerness to play (e.g., priority over other matters, thinking about gaming when not playing, and feeling bad if not playing). Factor 3 (4 items): Perceived unwillingness to stop playing without completion of gaming tasks that contains related items describing such tendencies (e.g., repeated attempts when not successful, uncomfortable feelings about unfinished goals, and strong urges to complete a goal as soon as possible). The three factors showed eigenvalues of 11.2, 1.3, and 1.1 and explained 46.5%, 5.4%, and 4.7% of the total variance (56.6%), respectively. All items presented factor loadings > 0.50 (Table 3). The 15 items were included in the CFA, which showed satisfactory goodness-of-fit to confirm a 3-factor structure (Chi-square/df = 2.900; NFI = 0.904; CFI = 0.934; RMSEA = 0.070). The standardized path estimates ranged from 0.46 to 0.79 (*p* < 0.001) (Table 3); the correlations among the three factors ranged from 0.64 to 0.68. C-RIGCS was hence constructed.

### 3.4. Analysis of C-RIGCS and Its Subscales

The mean scores of the overall scale and its three subscales were 16.8 (SD = 11.7, range: 0–60), 7.2 (SD = 6.0, range: 0–28), 3.7 (SD = 3.5, range: 0–16), and 5.9 (SD = 3.8, range: 0–16), respectively. No floor effect was noticed, except for the perceived urges for playing Internet games subscale (overall scale: 6.5%; perceived rewards of Internet gaming: 11.7%; perceived urges for playing Internet games: 21.6%; perceived unwillingness to stop playing without completion of gaming tasks: 6.8%). Similarly, no ceiling effect was observed as only 0.7% to 1.7% reported maximum total or subscale scores (see Table 4). The Cronbach’s alpha values were 0.91, 0.87, 0.81, and 0.72 for the overall C-RIGCS scale and its three subscales, respectively. The test-retest ICC coefficients were 0.79, 0.80, 0.75, and 0.71 for the overall C-RIGCS and its three subscales, respectively (see Table 5). The item-scale correlation coefficients ranged from 0.52 to 0.72 (all *p* < 0.001); the item-subscale correlation coefficients ranged from 0.66 to 0.81 (all *p* < 0.001). All correlation coefficients between the individual items and their respective subscale were higher than those between the items and the other two subscales (see Table 5).

### 3.5. External Correlations

The overall scale and its three subscales were all positively correlated with IGD (overall scale: r = 0.38; perceived rewards of Internet gaming: r = 0.32; perceived urges for playing Internet games: r = 0.40; perceived unwillingness to stop playing without completion of gaming tasks: r = 0.30; all *p* < 0.001) and average gaming time per week in the past month (overall scale: r = 0.47; perceived rewards of Internet gaming: r = 0.42; perceived urges for playing Internet games: r = 0.44; perceived unwillingness to stop playing without completion of gaming tasks: r = 0.38; all *p* < 0.001). Similarly, the overall C-RIGCS and its three subscales were all positively correlated with the single item that assessed the perception of Internet gaming as the primary source of self-esteem (overall scale: r = 0.50; perceived rewards of Internet gaming: r = 0.51; perceived urges for playing Internet games: r = 0.40; perceived unwillingness to stop playing without completion of gaming tasks: r = 0.40; all *p* < 0.001) and the single item that assessed the perception of Internet gaming as the primary source of social acceptance (overall scale: r = 0.51; perceived rewards of Internet gaming: r = 0.53; perceived urges for playing Internet games: r = 0.40; perceived unwillingness to stop playing without completion of gaming tasks: r = 0.39; all *p* < 0.001). In addition, the overall scale and its three subscales were all positively correlated with impulsivity (overall scale: r = 0.35; perceived rewards of Internet gaming: r = 0.31; perceived urges for playing Internet games: r = 0.35; perceived unwillingness to stop playing without completion of gaming tasks: r = 0.27; all *p* < 0.001) and negatively correlated with self-control (overall scale: r = −0.36; perceived rewards of Internet gaming: r = −0.29; perceived urges for playing Internet games: r = −0.43; perceived unwillingness to stop playing without completion of gaming tasks: r = −0.27; all *p* < 0.001), respectively (see Table 6).

### 3.6. Validation in the Subsample of Internet Gamers

We further applied CFA of the 3-factor model in the subsample of Internet gamers (n = 493). The resulting model fit was also satisfactory (Chi-square/df = 3.511, CFI = 0.953, NFI = 0.936, RMSEA = 0.058). There were correlations between the factor scores and the six external variables in the subsample of Internet gamers; the findings were consistent with those of the overall sample, both in significance and directions, with absolute values of the correlation coefficients ranging from 0.31 to 0.49 (all *p* < 0.001). The results are not presented in the tables.

## 4. Discussion

The present study detected relatively high IGD prevalence of about 11% among all participants and about 16% among Internet gamers. There is a need to reduce IGD among Chinese adolescents. It is important to understand various domains of maladaptive cognitions about Internet gaming and their relationships with IGD, as such cognitions are potentially modifiable and understanding on such cognitions may facilitate development of effective behavioral interventions for reduction of IGD. The findings of this study contribute to that end as all the three newly identified cognitive factors of C-RIGCS were significantly associated with IGD. The two constructs of perceived urges for playing Internet games and perceived unwillingness to stop playing without completion of gaming tasks match with the first dimension of maladaptive gaming-related cognitions that refers to distorted or irrational cognitions promoting and maintaining excessive or pathological gaming [6,7], while the construct of perceived rewards of Internet gaming fits into the second dimension of maladaptive gaming-related cognitions that refers to potentially harmful beliefs about the self in relation to gaming [11]. Comparisons of the strengths of associations between different domains of cognitions of Internet gaming and IGD can further provide insights on the relative contributions of various types of cognitions on development of IGD, and guide design of effective intervention contents. In our case, cognitions about perceived urges for playing Internet games showed the strongest positive associations with IGD symptoms and gaming time, compared to the subscales of perceived rewards of Internet gaming and perceived unwillingness to stop playing without completion of gaming tasks. Future interventions may emphasize such cognitions.

As a prerequisite of designing interventions to modify cognitions, it is warranted to develop measurement tools for assessment of domains of cognitions related to Internet gaming in a comprehensive manner. Development of the original IGCS was a good starting point, as it was carefully derived from a systematic review and has been applied to design a potentially effective pilot intervention study that reduced IGD symptoms. The scale is, however, new and has not been subjected to cross-cultural validation. The CFA of the present study did not confirm the 4-factor structure of the original IGCS, which has not been subjected to CFA, although some favorable psychometric properties (e.g., internal reliability and external validity) were reported [17]. The lack of goodness-of-fit in the present study might be due to cultural and contextual differences. For instance, the level of tangibility and nature of negative consequences of Internet gaming may differ across countries; investigation of the differences is beyond the scope of this study. Nevertheless, some inferior psychometric properties of the original IGCS were also observed. For instance, the correlations among the four subscales of the original IGCS were very high (0.88 to 0.98), implying substantially overlapping constructs. For instance, social acceptance may contribute to self-esteem, while gaining self-esteem/social acceptance via Internet gaming may lead to perceptions on maladaptive rules. Relatedly, although the 24 items of the original IGCS were grouped into four domains according to similar contents/meanings and the principles of standard cognitive conceptualization [32], some items correlated strongly with multiple subscales of the original IGCS, as seen from the double loading of nine items in the EFA in this study.

We hence re-analyzed the 24 items in two relatively large split sub-samples and identified a new 3-factor structure by EFA (perceived rewards of Internet gaming, perceived urges for playing Internet games, and perceived unwillingness to stop playing without completion of gaming tasks) to form C-RIGCS, which is an improvement over the original 4-factor structure of IGCS. First, it was confirmed by CFA. Second, nine double-loaded items were removed to form the C-RIGCS; the correlations among the three factors became much lower than those among the original four factors; the three new factors thus showed clearer conceptual distinctions. Third, the modified C-RIGCS showed satisfactory psychometric properties, including internal consistencies (i.e., 0.70 or above for Cronbach’s alpha), test-retest reliability (i.e., 0.70 or above for ICC coefficients), and content validity (i.e., strong correlations between the items and their corresponding subscales but weaker correlations between the items and other subscales). No ceiling effect was noticeable. The perceived urges for playing Internet games subscale showed a slight floor effect, possibly due to the relatively high proportion of non-gamers (33.1% did not play Internet games in the past year) in the sample, whose potentially low gaming urges might have contributed to the observed floor effect.

Furthermore, the 3-factor model is conceptually different from the original 4-factor model. The new perceived rewards of Internet gaming factor included items of various subscales of the original IGCS. Although the different types of potential rewards (e.g., self-esteem, social acceptance, and tangible rewards) described by the original IGCS are conceptually different, Chinese adolescents may just perceive various types of rewards of Internet gaming as a single entity. The high Cronbach value of this subscale supports this conjecture. Perceived urges for playing Internet games and perceived unwillingness to stop playing before completion of gaming tasks were the other two factors identified in this study. It seems that urges to play may be associated with preoccupation with Internet gaming, loss of interest, and inability to control Internet gaming in other activities (DSM-5 criteria); perceived unwillingness to stop playing without completion may be associated with significant loss due to Internet gaming (DSM-5 criteria). Thus, the 3-factor structure may have some conceptual implications.

Besides, the three new subscales were all significantly associated with the six external variables. As expected, the new perceived rewards of Internet gaming factor showed stronger associations than the other two factors with the perceptions of Internet gaming being the primary sources of self-esteem and social acceptance. It is plausible that beliefs on rewards of Internet gaming was associated with reliance on Internet gaming as primary sources of gaining self-esteem and social acceptance. Likewise, the perceived urges for playing Internet games subscale showed stronger associations with both impulsivity and self-control than the other two factors; urges for Internet gaming may be signs of impaired self-control and impulsivity. Some degrees of convergent validity have hence been observed in these cases.

The findings may lead to some new research directions. Future studies should validate both the original version of IGCS and C-RIGCS in English and other languages to understand better the comprehensive structure of cognitions related to Internet gaming and their relationships with IGD. The inter-relationships among the three factors of C-RIGCS should also be studied, although it is beyond the scope of this study. For instance, the relationship between gaming for reward beliefs and IGD may be mediated by cognitions on urges/stopping. Future studies should also clarify differences in gaming-specific cognitions between Internet gamers and non-gamers. As the cognitions of adults and adolescents about Internet gaming are likely to differ, future studies should test validity and required modification of the C-RIGCS among adults.

The present study has several limitations. First, responses were self-reported and may involve reporting bias although the survey was anonymous. Second, the schools were not randomly selected and only seven schools in two major Chinese cities were involved; selection bias may occur. Third, about 1/3 of the participants did not play Internet games in the past 12 months. They also answered the questions as they might have played games previously, and they can also have beliefs about Internet gaming. It is a strength that our findings showed that the validation results among the gamer subsample were highly consistent with those derived from the overall study population. Lastly, although the original scale was based on a systematic review and a conceptual model, a number of items have been removed; the new analysis was hence based on EFA rather than the original model that did not show satisfactory goodness of fit.

## 5. Conclusions

The present study has validated a revised instrument that can be used to assess maladaptive cognitions specific to Internet gaming. It proposes a new three-factor model (i.e., perceived rewards of Internet gaming, perceived urges for playing Internet games, and perceived unwillingness to stop playing without completion of gaming tasks) that showed satisfactory psychometric properties. Its application may facilitate future research on the impact of gaming-specific cognitions on IGD and develop effective intervention programs for reducing IGD among adolescents. It can potentially be applied to both the general adolescent population and among adolescent Internet gamers. Based on the findings, we suggest the use of C-RIGCS to measure maladaptive gaming cognitions among adolescents in China.

## Figures and Tables

**Table 1:** Characteristics of participants in the present study.

Variables	n	%
Total	755	100
Background variables		
Gender		
Female	354	46.9
Male	401	53.1
Household income level as compared to their classmates’		
Much higher/higher	171	22.8
Moderate	493	65.6
Lower/Much lower	87	11.6
Self-reported academic performance		
Above average	243	33.6
Average	325	44.9
Below average	156	21.5
Internet gaming-related variables/scales		
Ever played Internet games in the past 12 months		
No	244	33.1
Yes	493	66.9
Internet gaming disorder		
No	667	88.3
Yes	88	11.7
Average gaming time per week in the past month		
Nil (Non-gamers)	244	33.7
<2 h	219	30.3
2~<6 h	154	21.3
6~<10 h	51	7.1
≥10 h	55	7.6
Internet gaming is the primary source of my self-esteem		
Extremely disagree/agree	554	73.7
Neutral	140	18.6
Agree/extremely agree	58	7.7
Internet gaming is the primary source of my social acceptance		
Extremely disagree/agree	551	73.6
Neutral	148	19.8
Agree/extremely agree	50	6.7
	**Mean**	**SD**
Impulsivity	22.8	7.9
Self-control	44.2	7.7

**Table 2:** Confirmatory factor analysis of the original Internet Gaming Cognition Scale.

Items	β
Factor 1: Overvaluation	
IGC 3. Rewards in Internet games are as important to me as anything else in my life.	0.61
IGC 4. When my game character achieves something, I feel like I have achieved that too.	0.70
IGC 6. Playing Internet games has many other benefits in my life.	0.69
IGC 13. I find myself thinking about video games when I am not playing.	0.67
IGC 14. I spend time planning or thinking about the next thing I need to do in a game.	0.72
Factor 2: Maladaptive rules	
IGC 1. When I make mistakes, lose progress, or fail in a game, I must reload and try again.	0.56
IGC 2. It would be a waste to stop playing a game I have already invested so much time and energy in.	0.46
IGC 8. When I have a goal or objective in a video game, I must complete it as soon as possible.	0.65
IGC 9. I prioritize video games before doing something else, e.g., homework or chores.	0.65
IGC 10. I tell myself ‘just a few more minutes’ when I play a game, but then play much longer.	0.64
IGC 11. I feel uncomfortable thinking about my unfinished goals or objectives in video games.	0.71
IGC 15. I feel unsatisfied until I have done everything I want to do in a video game.	0.75
IGC 16. No amount of gaming time ever feels like “long enough”.	0.73
Factor 3: Gaming for self-esteem	
IGC 5. I tend to feel better after playing Internet games.	0.70
IGC 7. I would feel bad if I was not able to play Internet games.	0.65
IGC 12. I am proud of my gaming achievements.	0.75
IGC 20. I feel more in control when I play video games.	0.69
IGC 21. I would not be able to cope with stress in my life without video games.	0.64
IGC 23. If I complete or master an achievement, skill or goal in a video game, I feel good about myself.	0.77
Factor 4: Gaming for social acceptance	
IGC 17. People who do not play video games do not really understand an important part of who I am.	0.70
IGC 18. It is important to me that I am better at certain video games than other players.	0.72
IGC 19. Other players admire and respect my gaming achievements.	0.70
IGC 22. I feel safer and more comfortable playing a video game than in most other social situations.	0.71
IGC 24. When I succeed in a video game, players notice and respect me.	0.74

**Table 3:** Exploratory and confirmatory factor analyses of the Chinese version of revised Internet Gaming Cognition Scale.

Items	Factor Loading (EFA)			Path Estimate (CFA)	Factor in the Original Scale
	Factor 1	Factor 2	Factor 3	β	
Subscale 1: Perceived rewards of Internet gaming					
IGC 3. Rewards in Internet games are as important to me as anything else in my life.	**0.50**	0.25	0.36	0.61	OV
IGC 5. I tend to feel better after playing Internet games.	**0.61**	0.28	0.31	0.69	GE
IGC 6. Playing Internet games has many other benefits in my life.	**0.71**	0.21	0.25	0.71	OV
IGC 17. People who do not play video games do not really understand an important part of who I am.	**0.68**	0.38	0.12	0.68	GA
IGC 20. I feel more in control when I play video games.	**0.74**	0.10	0.29	0.70	GE
IGC 22. I feel safer and more comfortable playing a video game than in most other social situations.	**0.65**	0.27	0.23	0.70	GA
IGC 24. When I succeed in a video game, players notice and respect me.	**0.61**	0.19	0.33	0.74	GA
Subscale 2: Perceived urges for playing Internet games					
IGC 7. I would feel bad if I was not able to play Internet games.	0.36	**0.55**	0.28	0.77	GE
IGC 9. I prioritize video games before doing something else, e.g., homework or chores.	0.20	**0.75**	0.19	0.74	MR
IGC 10. I tell myself ‘just a few more minutes’ when I play a game, but then play much longer.	0.06	**0.66**	0.38	0.73	MR
IGC 13. I find myself thinking about video games when I am not playing.	0.29	**0.69**	0.21	0.72	OV
Subscale 3: Perceived unwillingness to stop playing without completion of gaming tasks					
IGC 1. When I make mistakes, lose progress, or fail in a game, I must reload and try again.	0.15	0.21	**0.64**	0.58	MR
IGC 2. It would be a waste to stop playing a game I have already invested so much time and energy in.	0.11	0.29	**0.51**	0.46	MR
IGC 8. When I have a goal or objective in a video game, I must complete it as soon as possible.	0.21	0.29	**0.70**	0.69	MR
IGC 11. I feel uncomfortable thinking about my unfinished goals or objectives in video games.	0.32	0.26	**0.57**	0.79	MR
Eigen value	11.2	1.3	1.1		
Cumulative % of Variance explained	46.5	51.9	56.6		

Abbreviations: EFA, exploratory factor analysis; CFA, confirmatory factor analysis; OV, overvaluing subscale; GE, gaming for self-esteem subscale; GA, gaming for social acceptance subscale; MR, maladaptive rules subscale. Bold: factor loadings > 0.50.

**Table 4:** Descriptive statistics for the subscales of the Chinese version of revised Internet Gaming Cognition Scale.

Items	Score	Mean ± SD	Floor Effect (%)	Ceiling Effect (%)
Overall scale	0–60	16.8 ± 11.7	6.5	0.7
Perceived rewards of Internet gaming	0–28	7.2 ± 6.0	11.7	0.7
Perceived urges for playing Internet games	0–16	3.7 ± 3.5	21.6	1.0
Perceived unwillingness to stop playing without completion of gaming tasks	0–16	5.9 ± 3.8	6.8	1.7

**Table 5:** Internal consistency, test–retest reliability, and item analysis of the Chinese version of revised Internet Gaming Cognition Scale.

	Cronbach’s Alpha		Item—Scale Scale Correlation	Item—Subscale Correlation	Item—Other Subscale Correlation
Items	Subscale	Total			
Overall scale	0.91	-	-	-	-
Factor 1: Perceived rewards of Internet gaming	0.87	-	-	-	-
IGC 3	0.86	0.94	0.63 ***	0.66 ***	0.45 ^a^***/0.50 ^b^***
IGC 5	0.85	0.94	0.72 ***	0.75 ***	0.55 ^a^***/0.54 ^b^***
IGC 6	0.84	0.93	0.72 ***	0.78 ***	0.51 ^a^***/0.51 ^b^***
IGC 17	0.85	0.93	0.69 ***	0.74 ***	0.52 ^a^***/0.46 ^b^***
IGC 20	0.85	0.93	0.67 ***	0.77 ***	0.42 ^a^***/0.48 ^b^***
IGC 22	0.85	0.93	0.70 ***	0.76 ***	0.52 ^a^***/0.48 ^b^***
IGC 24	0.85	0.93	0.69 ***	0.75 ***	0.46 ^a^***/0.51 ^b^***
Factor 2: Perceived urges for playing Internet games	0.81	-	-	-	-
IGC 7	0.76	0.93	0.71 ***	0.79 ***	0.59 ^a^***/0.54 ^c^***
IGC 9	0.76	0.93	0.65 ***	0.79 ***	0.51 ^a^***/0.47 ^c^***
IGC 10	0.76	0.93	0.65 ***	0.81 ***	0.48 ^a^***/0.51 ^c^***
IGC 13	0.75	0.93	0.67 ***	0.80 ***	0.54 ^a^***/0.49 ^c^***
Factor 3: Perceived unwillingness to stop playing without completion of gaming tasks	0.72	-	-	-	-
IGC 1	0.66	0.93	0.60 ***	0.73 ***	0.47 ^b^***/0.42 ^c^***
IGC 2	0.72	0.93	0.52 ***	0.68 ***	0.36 ^b^***/0.37 ^c^***
IGC 8	0.61	0.93	0.69 ***	0.79 ***	0.55 ^b^***/0.51 ^c^***
IGC 11	0.64	0.93	0.72 ***	0.75 ***	0.60 ^b^***/0.57 ^c^***

Note: ***, *p* < 0.001; ^a^, Pearson coefficient for the correlation analyses between factor 1 and factor 2; ^b^, Pearson coefficient for the correlation analyses between factor 1 and factor 3; ^c^, Pearson coefficient for the correlation analyses between factor 2 and factor 3.

**Table 6:** Correlations between Internet gaming cognitions and external variables.

	IGD	Gaming Time	Internet Gaming Is the Primary Source of Self-Esteem	Internet Gaming Is the Primary Source of Social Acceptance	Impulsivity	Self-Control
	r_p_	r_s_	r_s_	r_s_	r_p_	r_p_
Overall scale	0.38 ***	0.47 ***	0.50 ***	0.51 ***	0.35 ***	−0.36 ***
Perceived rewards of Internet gaming	0.32 ***	0.42 ***	0.51 ***	0.53 ***	0.31 ***	−0.29 ***
Perceived urges for playing Internet games	0.40 ***	0.44 ***	0.40 ***	0.40 ***	0.35 ***	−0.43 ***
Perceived unwillingness to stop playing without completion of gaming tasks	0.30 ***	0.38 ***	0.40 ***	0.39 ***	0.27 ***	−0.27 ***

Note: r_p_, Pearson correlation coefficient; r_s_, Spearman correlation coefficient; ***, *p* < 0.001.
